# Roles for retrotransposon insertions in human disease

**DOI:** 10.1186/s13100-016-0065-9

**Published:** 2016-05-06

**Authors:** Dustin C. Hancks, Haig H. Kazazian

**Affiliations:** Eccles Institute of Human Genetics, University of Utah School of Medicine, Salt Lake City, UT USA; McKusick-Nathans Institute of Genetic Medicine, The Johns Hopkins School of Medicine, Baltimore, MD USA

**Keywords:** Retrotransposon, LINE-1, Alu, SVA, LINE-1, Disease, Retrotransposition, Autoimmunity, Cancer

## Abstract

Over evolutionary time, the dynamic nature of a genome is driven, in part, by the activity of transposable elements (TE) such as retrotransposons. On a shorter time scale it has been established that new TE insertions can result in single-gene disease in an individual. In humans, the non-LTR retrotransposon Long INterspersed Element-1 (LINE-1 or L1) is the only active autonomous TE. In addition to mobilizing its own RNA to new genomic locations via a “copy-and-paste” mechanism, LINE-1 is able to retrotranspose other RNAs including Alu, SVA, and occasionally cellular RNAs. To date in humans, 124 LINE-1-mediated insertions which result in genetic diseases have been reported. Disease causing LINE-1 insertions have provided a wealth of insight and the foundation for valuable tools to study these genomic parasites. In this review, we provide an overview of LINE-1 biology followed by highlights from new reports of LINE-1-mediated genetic disease in humans.

## Background

### A brief history

Transposable elements (TEs) are pieces of nucleic acid that encode the inherent ability to mobilize from one genomic location to another. This ability to “jump” is mediated by element-encoded proteins such as DNA transposase or reverse transcriptase. These TEs are referred to as autonomous. In other instances, non-coding TEs -typically referred to as non-autonomous- contain sequence features (e.g. sequence motifs, RNA structural elements), which are recognized by autonomous TE proteins that ultimately result in *trans*-mobilization of these sequences. Collectively, autonomous and non-autonomous transposable elements often comprise greater than 50 % of genomic real estate in mammals. For humans, approximately two-thirds of our genome can be annotated as TE-derived [1–6]; however, it is likely that the actual percentage is greater but due to sequence decay no sequence identity can be assigned.

Almost 70 years ago, Barbara McClintock laid the foundation for TE research with her initial work and discoveries in maize of what she termed “controlling elements [7].” Since that time, several discoveries have been made leading to an active research community investigating the impact of transposable elements on the human genome and their role in disease. Although work by Britten and Davidson in the 1960s provided hints that the human genome was largely repetitive [8, 9], it wasn’t until the Human Genome Project [4–6] that the true origin and extent of the repeats in our genome became evident. The initial human genome draft sequence estimated that roughly 45 % of our genomic sequence is derived from TE sequence. The Human Genome and other genome projects [1, 3, 6] significantly transformed TE biology by providing the ability to answer questions including 1) Which TEs have been the most active?, 2) Where are specific TEs maintained in the genome?, 3) Which elements and how many have been recently active?

A pivotal transformation in TE biology occurred less than 10 years after the publication of the Human Genome Project. Next-generation sequencing has empowered researchers to interrogate longstanding and previously intractable questions regarding TE biology [7, 10, 11]. Examples include the frequency and location of new insertions and the contribution of TEs to gene regulation genome-wide at an unprecedented resolution [8, 9, 12, 13]. New studies will likely unveil novel ways by which these selfish genetic elements may actually be altruistic or even co-opted by the host genome [14] along with new insights into mechanisms by which they can cause disease. Here we provide an update of human TE biology, with a specific emphasis on LINE-1-mediated retrotransposition and disease-causing insertions.

## Human transposable elements

**Fig. 1:**
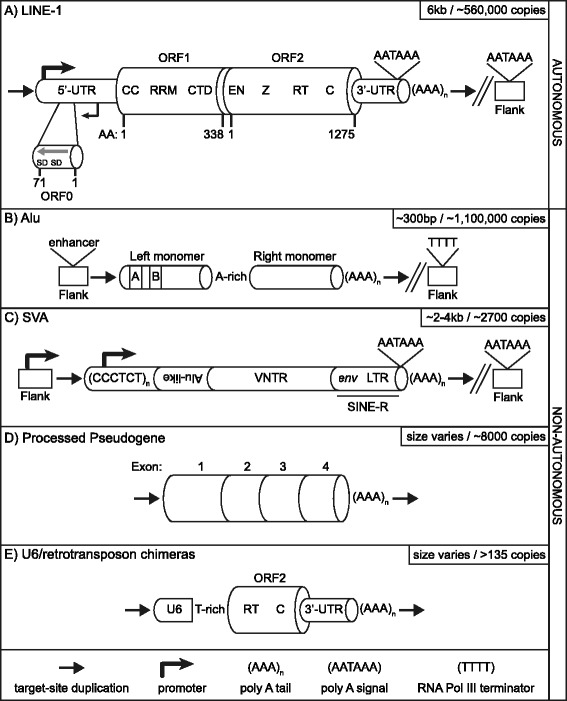
Retrotransposons active in humans. **a** An autonomous active LINE-1. A full-length LINE-1 ~ 6 kb in length is shown [36, 41, 239]. LINE-1 encodes three proteins, two of which (ORF1p and ORF2p) are absolutely required for retrotransposition *in cis* [42, 146]. Currently, the role for ORF0p is unclear [60]; interestingly, it may form fusion proteins with downstream coding sequences by utilizing internal splice donor sites (SD) [60]. LINE-1 transcription is driven from its own promoter (big black bent arrow) [53, 54] located in the 5′-UTR. The 5′-UTR also encodes a weaker antisense promoter (ASP, small black bent arrow) [59]. It has been postulated that the LINE-1 ASP in conjunction, with splice acceptors located on the antisense strand of LINE-1, may contribute to new gene formation via a mechanism termed “gene-breaking [240].” Termination of LINE-1 transcription is mediated by a polyA signal (AATAAA) located in the 3′-UTR. Occasionally, transcription proceeds past the internal polyA signal and terminates at a downstream one [139, 241]. Such chimeric transcripts, if retrotransposed, may result in 3′-transductions [42, 62–64, 176]. Majority of insertions end in a polyA tail (AAA_n_) of variable length [37]. In addition, most insertions are characterized by flanking target-site duplications (4-20 bp in length, black horizontal arrows) [35]. CC-coiled coiled domain [47], RRM-RNA recognition motif [44], CTD-C-terminal domain, EN-endonuclease [51], Z domain [242], RT-reverse transcriptase [52], C-cysteine-rich. AA-amino acid. **b** The Alu SINE. Alus are small Pol III transcribed RNAs derived from 7SL RNA [243]. An Alu element consists of a left and right monomer, which are derived from an ancient duplication event, separated by an internal A-rich sequence. Alus contain their own transcriptional signals, an A and B box located in the left monomer. Efficient Alu transcription requires a strong enhancer element in the upstream flanking sequence [103, 104]. Transcription termination of an Alu typically occurs at a Pol III terminator (TTTT) located in the downstream flanking sequence [244]. Similar to LINE-1, Alu insertions end in a polyA tail and are flanked by a target-site duplication. **c** A canonical SINE-VNTR-Alu (SVA) element consisting of its primary domains: CCCTCT hexamer, Alu-like, VNTR, SINE-R derived from the *env* gene and right LTR from a HERV-K is shown [126]. SVA transcription can initiate upstream (black bent arrow) or in the CCCTCT hexamer (black bent arrow) [126, 127]. Like LINE-1, SVA transcription typically terminates at its own [127] or a downstream polyA signal [24, 65]. **d** A processed pseudogene (PP) is shown. Note the lack of introns and the presence of a target-site duplication and a 3′-polyA tail similar to LINE-1, Alu, and SVA. **e** U6 chimera insertion. A U6 snRNA fused with the 3′-end of an LINE-1 sequence formed by “template-switching” [84, 140, 144] is shown. Although the site where ORF2p switches templates varies across the U6 chimera insertions, the junction where the two sequences are joined is typically T-rich [144]

TEs are historically subdivided into two major classes defined by their mobilization intermediate. Class I TEs, also known as retrotransposons, encompass elements that move via a “copy-and-paste” mechanism involving an RNA intermediate [15, 16], while Class II TEs, referred to as DNA transposons, represent TEs that mobilize by a “cut-and-paste” mechanism. DNA transposons are currently thought to be transpositionally inactive in most mammals with bats being the exception [17, 18]; however, several genes in the human genome are derived from DNA transposons [6]. Three of these genes (recombination activating gene 1 (*RAG1*) [19], PiggyBac transposable element-derived protein 5 (*PGBD5*) [20], and THAP domain containing 9 (*THAP9*) [21])) are evolutionarily conserved and can carry out DNA transposition in cell culture or perform reactions reminiscent of DNA transposition. In contrast, retrotransposons (Fig. 1) remain quite active in humans [22–24]; any two human beings differ on average by ~285 different LINE-1 insertions [25].

Retrotransposons can be further subdivided into two subclasses: those with Long-Terminal Repeats (LTR) and those without (non-LTR). LTR elements, also known as endogenous retroviruses (ERVs), comprise ~8 % of the human genome [6]. Many of these elements lack a majority of the viral genes and exist only as single LTRs, often referred to as solo LTRs. Similar to DNA transposons, LTR elements are thought to be inactive in the human lineage, although rare polymorphic ERVs in the human population indicate that mobilization has occurred following the human-chimpanzee divergence [26–28]. Very recently, several unfixed HERV-K elements were identified across human genomes including an intact insertion that still may be infectious [29]. In contrast, ERVs have been active recently in the chimpanzee and gorilla lineages [30]. Most ERVs are speculated to be exogenous viruses that integrated into the host germline in the distant past [31, 32]. There is some evidence that endogenous viral elements (EVEs) may have escaped the cell by acquiring a functional envelope gene and that these genetic elements are the ancestors of modern-day retroviruses [33]. Certain hints already exist, but as more genomes are analyzed one might predict that formation of infectious viruses from endogenous elements followed by re-endogenization of exogenous elements might be more common than previously appreciated [34].

### LINE-1

Long INterspersed Element-1 (LINE-1 or L1), a non-LTR element, is the only active autonomous TE in man. Despite the fact that the human genome contains more than 500,000 LINE-1 sequences, most are inactive due to rearrangements, point mutations, and 5′-truncation [6, 35–37]. Only a small subset, 80-100 LINE-1 s, are thought to be active in any given individual [38, 39], with each set of active elements differing between individuals [40]. An active LINE-1 residing in the genome is 6 kb in length [41] (Fig. 1a) contains a 5′- and 3′-UTR, encodes two proteins (i.e. bicistronic), ORF1p and ORF2p, separated by a 63 bp inter-ORF spacer and ends in a long polyA tail. Cell culture retrotransposition assays indicate that both proteins are absolutely required for LINE-1 mobilization in *cis* [42]. ORF1p is a ~40 kDa protein [43] with RNA binding [44, 45] and chaperone activities [46]. Although structural analysis and biochemical studies [47] have revealed that ORF1p forms a series of trimers with nucleic acids [48, 49] via rapid polymerization mediated by coiled-coiled domain interactions, its precise function remains poorly understood; however, new work indicates that phosphorylation of ORF1p is required for retrotransposition [50]. ORF2p is a 150 kDa protein with endonuclease (EN) [51] and reverse transcriptase (RT) [52] activities.

LINE-1 is transcribed from its own promoter [53] located in the ~900 bp 5′UTR presumably by RNA Pol II. LINE-1 RNAs are thought to be capped as evidenced by untemplated guanosines at the 5′-end of full-length genomic insertions [54]. Several transcription factors have been implicated in LINE-1 transcription including ying yang 1 (YY1) [55], T-cell factor/lymphoid enhancer factor (TCF/LEF) [56], p53 [57], and runt related transcription factor 3 (RUNX3) [58]. LINE-1 also contains an antisense promoter in the 5′-UTR [59]. Recently, a novel ORF termed *ORF0*, which is 70 amino acids in length, was identified on the antisense strand of primate LINE-1 5′UTRs [60]. As *ORF0* has two splice donor sites, *ORF0* has the ability to form fusion proteins with downstream exons [60]. Interestingly, overexpression of ORF0p *in trans* results in a 41 % increase in engineered LINE-1 retrotransposition in cell culture [60]. Future research will reveal the role of ORF0p and whether functional homologs have been independently derived in other species.

**Fig. 2:**
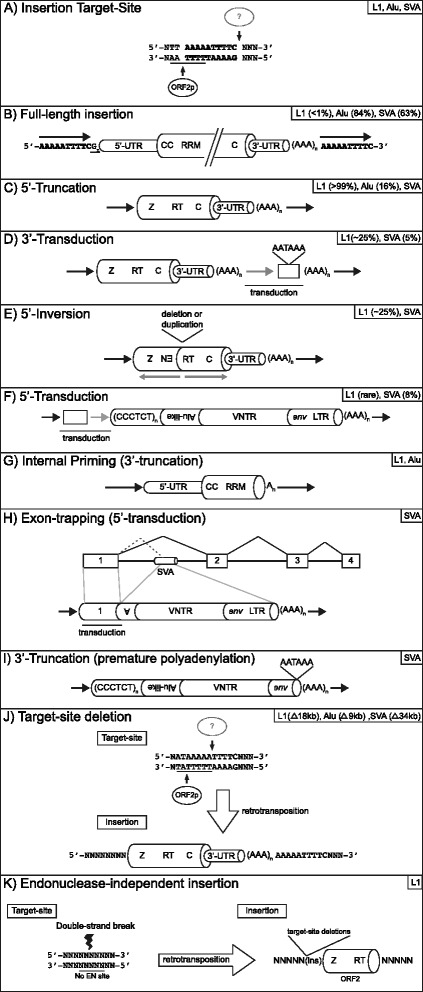
Anatomy of retrotransposon insertions. A variety of structures for retrotransposon insertions (**a**-**k**) identified by genomic studies, cell culture retrotransposition assays, and disease-causing insertions that have been reported is shown. Reported frequencies, either from genomic analysis or cell-culture retrotransposition assays, for each structure is located in the upper right hand corner of each panel. If no frequency data has been reported only the element’s name is shown. These structures have provided key insights into the mechanism of target-primed reverse transcription [77], retrotransposon transcript structure [127], and the mechanism by which LINE-1-mediated retrotransposition events contribute to genome evolution [62]. A) LINE-1 target-site. Most insertions occur at asymmetric AT-rich sequences [6, 37, 51, 86]. The first step of TPRT is cleavage of the bottom-strand by ORF2p endonuclease activity at a motif resembling 5′-TTTT/AA-3′ [245]. The nuclease responsible for top-strand cleavage is currently unknown. The nature of the staggered cleavage events generates a target-site duplication (TSD, sequence in bold). **a** TSD (black horizontal arrows) is used to define the boundaries of an insertion and considered a hallmark of LINE-1-mediated retrotransposition events. **b** Full-length insertion. It is generally accepted that in order for an element to be retrotransposition-competent it must be full-length. **c** 5′-truncated insertions. Most LINE-1 s in the human genome are grossly truncated at their 5′-end [6, 36, 37]. In contrast, most Alus [243] and SVA elements are full-length [123, 127]. To date, no consensus sequence has been identified in LINE-1 or SVA insertions regarding the mechanism of 5′-truncation. However, a new report implicates stem-loop structures as a factor driving 5′-truncation in recent Alu insertions [114]. **d** 3′-transduction. Although the first report of a 3′-transduction was an LINE-1 insertion into the *dystrophin* gene resulting in Duchenne’s muscular dystrophy in 1994 [176], it would be several years before the significance of this chimeric insertion was uncovered. Several years later, as one of the first insights gained from insertions recovered from cell-culture retrotransposition assays, it was reported that LINE-1 frequently bypassed its own polyA signal (AATAAA) in favor of a downstream one (AATAAA) [42]. Subsequently, elegant experimental analysis revealed that utilizing a downstream polyA signal could result in LINE-1-mediated exon-shuffling [62]. An insertion containing a 3′-transduction will typically contain two homopolymer stretches (AAA_n_) and contain the 3′-TSD from the source locus (gray horizontal arrow) as part of the transduced sequence. Notably, insertions containing serial 3′-transductions have been reported and can be used to track the evolutionary history of an element [246]. **e** 5′-end inversions. Another hallmark of LINE-1-mediated retrotransposition events is the inversion of the 5′-end (gray horizontal arrow) of the retrotransposon sequence [35]. Small indels are typically identified at the inversion breakpoint [88]. Inversions have only been reported for LINE-1 s, SVAs, and processed pseudogenes [196]. 5′-end inversion is presumed not to occur for Alus due to their short length. It has been hypothesized that a phenomenon referred to as twin-priming may account for the frequent inversions associated with LINE-1-mediated retrotransposition events [88]. **f** 5′-transduction. In some instances, LINE-1 [82] or SVA transcription [126, 127] may initiate upstream of the internal promoter generating a chimeric transcript. Retrotransposition of this sequence results in duplication of the sequence 5′- of the source locus at a new genomic location. It has been speculated that 5′-transductions are relatively common for SVA elements due to their weaker internal promoter compared to LINE-1, which has a very strong internal promoter, where only a handful of 5′-transductions have been reported [82]. **g** Internal priming. Occasionally following bottom-strand cleavage, internal A-rich sequences upstream in the retrotransposon RNA may basepair with the T-rich overhang at the target-site instead of the 3′-polyA tail, followed by first-strand cDNA synthesis by ORF2p [247–249]. These insertions can be deemed a type of 3′-truncation. **h** Exon-trapping. Retrotransposons are dispersed throughout the genome including intronic sequence. LINE-1, Alu, SVA all have been reported to contain numerous splice sites and be incorporated into the transcriptome [105, 127, 128, 134, 250]. Interestingly, LINE-1 internal splicing can generate a transcript lacking ORF1 but maintaining a functional ORF2 [251]. In some instances, at least for SVA, retrotransposition of chimeric transcripts containing upstream exons may occur [127, 128, 132]. Notably, SVA itself is thought to have originated from alternative splicing from genomic repeats [126] and SVA-related elements (e.g. LAVA, PVA) appear to have acquired distinct 3′-domains via splicing in gibbons [125, 135, 136, 138]. I) 3′-truncation. Premature polyadenylation using either canonical or non-canonical polyadenylation sites results in LINE-1 or SVA RNAs lacking 3′-sequence [127, 252]. If this RNA is retrotransposed, it will result in a 3′-truncated insertion. Consistent with the dispensability of SVA domains [130], 3′-truncations may be more frequent for SVA compared to LINE-1. In principle, 3′-truncated LINE-1 RNAs containing ORF1 coding sequence might be actively retrotransposed as in the case of ORF1 *mNEOi* in cell culture [144] and the presence of half-LINE-1 (HAL1) insertions in mammalian genomes [253]. **j** Target-site deletion. Another surprise from cell culture retrotransposition assays was the discovery of large deletions associated with new retrotransposition events [82, 83]. Genomic deletions up to 1 MB have been associated with LINE-1 mediated retrotransposition events in vivo [153]. These insertions occur at a LINE-1 EN cleavage site, are generated by ORF2 reverse-transcriptase activity, and end in a 3-polyA tail. Currently, the mechanism driving 5-targe-site deletions is unclear; yet, it is tempting to speculate that chromatin looping along with cleavage by LINE-1 or another nuclease may play important roles [82, 83]. **k** Endonuclease-independent (EN_i_) insertion. En_i_ insertions were discovered by the Moran lab when carrying out retrotransposition assays in different Chinese Hamster Ovary (CHO) cell lines lacking key DNA repair factors [213]. Frequent retrotransposition was observed for an engineered LINE-1 element construct, with a catalytically inactive EN, in these cells but not HeLa cells. Characterization of recovered insertions revealed LINE-1 integration at genomic sites not resembling the LINE-1 EN consensus cleavage site. In addition, the insertions were typically truncated at both the 5′-and 3′-ends [213]. These data suggest that LINE-1 can serve as a “molecular band-aid” [254] at double-stranded DNA breaks [213–215] and that LINE-1 s lacking a functional EN domain may be able to retrotranspose in certain contexts. Building on these studies it was later reported that LINE-1 s can also integrate at dysfunctional telomeres in an endonuclease-independent manner [216]

Transcription of LINE-1 is terminated by an internal weak polyA signal (AATAAA) [42, 61, 62] present in the ~200 bp 3′-UTR. Frequently, LINE-1 transcription will read through its polyA signal in favor of a polyA signal located downstream of the genomic LINE-1 [62–64]. This downstream non-LINE-1 sequence is frequently retrotransposed to new genomic locations, a phenomena referred to as 3′-transduction (Fig. 2). 3′-transductions are an additional mechanism by which LINE-1 contributes to genomic expansion and a means to shuffle protein-coding exons throughout the genome [62, 65].

Following transcription from a genomic locus, the LINE-1 RNA is transported to the cytoplasm for protein translation and LINE-1 ribonucleoprotein (RNP) assembly. Although, the exact nature of LINE-1 ORF1p and ORF2p translation is not entirely resolved, significant insight comes from application of the cell culture retrotransposition assay. This work suggests that ORF2p is translated via an unconventional mechanism involving translation termination of ORF1 and reinitiation [66]. Surprisingly, this study demonstrated that the codon for any amino acid could serve as the +1 codon for ORF2p.

The next step in the LINE-1 lifecycle is RNP assembly [67]. While the number of ORF1p trimers is thought to be several, the number of ORF2p molecules in an active LINE-1 RNP is unknown but its abundance is thought to be significantly less when compared to ORF1p in the RNP [68]. In vitro analyses of non-LTR retrotransposon integration predict that at least 2 molecules of ORF2p are present in any given retrotranspositionally-competent (RC) LINE-1 RNP [69]. In addition, a new study has reported that the polyA tail of LINE-1 RNA is required in *cis* for formation of a RC-RNP presumably by serving to recruit ORF2p to the RNP [70]. Similarly, the polyA tail of Alu is also required for reverse transcription [70, 71]. Thus, the basal LINE-1 RNP contains ORF1p trimers, ORF2p, and the LINE-1 RNA. An active area of current research involves determining other components of the LINE-1 RNP, specifically which cellular RNAs [72] and non-LINE-1 proteins [73–76] are present.

LINE-1 insertions occur via a coupled reverse-transcription integration mechanism referred to as target-primed reverse-transcription (TPRT) [77, 78]. TPRT has been characterized in great detail biochemically by Eickbush and colleagues using the *Bombyx mori* non-LTR R2 element as a model. Although R2 differs from LINE-1 in that it only encodes one ORF, this ORF contains endonuclease [79] and reverse transcriptase activities [77]. How LINE-1 identifies a genomic neighborhood for integration remains of great interest. It is highly probable that chromatin states [80] and perhaps protein-protein interactions with nuclear factors dictate target-site preference.

The LINE-1 integration target-site (Fig. 2a) is determined by the ORF2p-encoded endonuclease [51, 81]. Biochemical [51], cell culture retrotransposition assays [42, 82–84], and genomic analysis [6] have revealed the LINE-1 EN consensus site to be 5′-TTTT/AA-3′ on the bottom-strand where “/” indicates the site of cleavage. The EN cleavage site is not absolute as variations are common and thus the site can better be defined as 5′-YYYY/RR-3′ where Y = pyrimidine and R = purine. The asymmetry of a pyrimidine followed by a purine at the cleavage site is almost always observed. See Table 1 for additional variations (YYRY/RR, YRYY/RR, etc).

**Table 1:** Retrotransposition events associated with human disease

	Insertion	Gene	CHR	Reference	Disease	Subfamily	Size	polyA tail length	Truncation	Transduction (bp)	Strand	Exon/Intron/Mechanism	Target-site duplication (TSD)	L1 EN site (5′-TTTT/AA-3′)	Note
1	Alu	*ABCD1*	X	Kutsche et al. 2002 [255]	ALD	AluYb9	98	20	Y/5′TR	N	S	4.7 kb Deletion	No TSD	ATTT/GT	
2	Alu	*ATP7A*	X	Gu et al. 2007 [256]	Menkes Disease	AluYa5a2	282	89	N	N	AS	E	AAAAAGGACAGC	TTTT/AT	
3	Alu	*BTK*	X	Lester et al. 1997 [257]	XLA	AluY	N/A	N/A	N/A	N	AS	E	N/A	N/A	
4	Alu	*BTK*	X	Conley et al. 2005 [258]	XLA	AluY	281	74	N	N	S	E	AGAAATGTATGAGTAAGT	TTCT/AT	Same insertion site Conley et al. SVA
5	Alu	*CD40LG*	X	Apoil et al. 2007 [259]	HIGM	AluYb8	292	8	N	N	AS	E	AAAAATTTTC	TTTT/AT	
6	Alu	*CLCN5*	X	Claverie-Martin et. al. 2003 [260]	Dent’s Disease	AluYa5	281	50	N	N	S	E	AGAAAATGCTCGAAAGA	TTCT/AT	
7	Alu	*CTRC*	1	Masson et. al. 2013 [160]	Chronic pancreatitis	Alu	31	11	Y/5′TR	N	AS	53.9 kb Deletion	N/A	TCTT/AT	Deletes entire CTRC and ELA2A genes
8	Alu	*PKLR*	1	Lesmana et. al. 2015 [159]	Severe Hereditary Nonspherocytic Hemolytic Anemia	Yb8	288	70	N	N	S	E	AAGATCATCAGCAAA	TCTT/GA	consanguinity, consensus Yb8
9	Alu	*FVIII*	X	Sukarova et. al. 2001 [261]	Hemophilia A	AluYb8	290	47	N	N	AS	3 nt Deletion	No TSD	TTTC/AT	
10	Alu	*FVIII*	X	Ganguly et. al. 2003 [262]	Hemophilia A	AluYb9	288	37	N	N	AS	I/Splicing	AAAAACCAACAGG	TTTT/AT	Consensus Yb9
11	Alu	*FVIII*	X	Green et. al. 2008 [263]	Hemophilia A	AluYb8	FL	N/A	N	N	AS	E	N/A		
12	Alu	*FIX*	X	Vidaud et al. 1993 [264]	Hemophilia B	AluYa5a2	244	78	Y/5′TR	N	S	E	AAGAATGGCAGATGCGA	TCTT/AA	Same insertion site as Wulff et al. Alu
13	Alu	*FIX*	X	Wulff et al. 2000 [265]	Hemophilia B	AluYa5a2	237	39	Y/5′TR	N	S	E	AAGAATGGCAGATGC	TCTT/AA	Same insertion site as Vidaud et al. Alu
14	Alu	*FIX*	X	Li et al. 2001 [266]	Hemophilia B	AluY	279	40	Y/5′TR	N	AS	E	AAGAAACTGGTCCC	TCTT/AA	
15	Alu	*GK*	X	Zhang et al. 2000 [267]	GKD	AluYc1	241	74	Y/5′TR	N	AS	I	AAAAAATAAG	TTTT/AA	
16	Alu	*IL2RG*	X	Lester et al. 1997 [257]	XSCID	AluYa5	N/A	N/A	N/A	N	AS	I	N/A	N/A	
17	Alu	*CRB1*	1	den Hollander et al. 1999 [268]	RP	AluY	244	70	Y/5′TR	N	AS	E	AAGAGTAAAGATGA	TCTT/GA	
18	Alu	*SERPINC1*	1	Beauchamp et al. 2000 [269]	Type 1 ATP	Alu	6	40	Y/5′TR	N	AS	1.4 kb Deletion	N/A	TTCT/AT	Shortest Alu insertion
19	Alu	*ALMS1*	2	Taşkesen et al. 2012 [270]	Alström syndrome	AluYa5	257	76	Y/5′TR	N	S	E	AAAAGCCTAGAGAA	TTTT/AA	
20	Alu	*MSH2*	2	Kloor et al. 2004 [271]	HNPCC	AluJ	85	40	Y/5′TR	N	S	E	N/A	N/A	Contains extra 99 nt 3′-of Alu, may be transduction or recombination
21	Alu	*MSH2*	2	Qian et al. 2015 [158]	Hereditary Cancer	N/A	N/A	N/A	N/A	N/A	N/A	E	N/A	N/A	Pan-cancer panel testing
22	Alu	*ZFHX1B*	2	Ishihara et al. 2004 [272]	MWS	AluYa5	281	93	N	N	S	E	AAAATTAAAACA	TTTT/AA	
23	Alu	*BCHE*	3	Muratani et al. 1991 [273]	Cholinesterase deficiency	AluYb9	289	38	N	N	S	E	AAAAATATTTTTTCC	TTTT/AA	
24	Alu	*CASR*	3	Janicic et al. 1995 [274]	FHH and NSHPT	AluYa5	280	93	N	N	AS	E	GAAAGCGTGAGCTGC	TTTC/AA	
25	Alu	*HESX1*	3	Sobrier et al. 2005 [275]	Anterior Pituitary Aplasia	AluYb8	288	30	N	N	S	E	AGAAAATGTCTTTAGA	TTCT/AA	
26	Alu	*OPA1*	3	Gallus et al. 2010 [276]	ADOA	AluYb8	289	25	N	N	AS	I/Splicing	AAAAATTTTAAAAAGTT	TTTT/AC	
27	Alu	*MLVI2*	5	Economou-Pachnis and Tsichlis 1985 [277]	Associated with leukemia	AluYa5	280	26	N	N	AS	I	GAAAATGT	TTTC/AT	
28	Alu	*APC*	5	Halling et al. 1999 [278]	Hereditary desmoid disease	AluYb8	278	40	Y/5′TR	N	S	E	AAGAATAATG	TCTT/AA	Same insertion site as Miki et al. L1
29	Alu	*APC*	5	Su et al. 2000 [279]	FAP	AluYb9	93	60	Y/5′TR	N	AS	I/Splicing	No TSD	TTTT/AA	1.6 kb intronic deletion
30	Alu	*APC*	5	Qian et al. 2015 [158]	Hereditary Cancer	N/A	N/A	N/A	N/A	N/A	N/A	E	N/A	N/A	Pan-cancer panel testing
31	Alu	*MAK*	6	Tucker et al. 2011 [280], Edwin Stone, personal communication	RP	AluYb8	281	57	N	N	AS	E	AAAGAAAAAA	CTTT/AA	Identified by exome resequence
32	Alu	*NT5C3*	7	Manco et al. 2006 [281], Leticia Ribeiro, personal communication	Chronic hemolytic anemia	Alu Ya5	281	36	N	N	S	E	AAGAATGGCAGATGG	TCTT/AA	
33	Alu	*CFTR*	7	Chen et al. 2008 [282]	Cystic Fibrosis	AluY	46	57	Y/5′TR	N	AS	E	AAGAATCCCACCTATAAT	TCTT/AA	
34	Alu	*CFTR*	7	Chen et al. 2008 [282]	Cystic Fibrosis	AluYa5	281	56	N	N	S	E	AATAGAAATGATTTTTGTC	TCTC/AT	3′-Processing of (5′-CTC-3′)
35	Alu	*EYA1*	8	Abdelhak et al. 1997 [283]	BOR syndrome	AluYa5	n/a	97,31	N/A	N	AS	E	AAAAAATAAATGTGTG	TTTT/AA	PolyA tail shortening between generations
36	Alu	*LPL*	8	Okubo et al. 2007 [284]	LPL deficiency	AluYb9	150	60	Y/5′TR	N	AS	2.2 kb Deletion	No TSD	TTTT/AA	
37	Alu	*CHD7*	8	Udaka et al. 2007 [285]	CHARGE syndrome	AluYa5/8	75	100	Y/5′TR	N	S	10 kb Deletion	No TSD	ATTT/AA	
38	Alu	*POMT1*	9	Bouchet et al. 2007 [286]	Walker Warburg syndrome	AluYa5	290	53	N	N	AS	E	AAAAAGAGATGTACTG	TTTT/AC	
39	Alu	*FGFR2*	10	Oldridge et al. 1999 [287]	Apert syndrome	AluYa5	283	69	N	N	AS	I/Splicing	AGAAAACAAGGGAAGCA	TTCT/AG	
40	Alu	*FGFR2*	10	Oldridge et al. 1999 [287]	Apert syndrome	AluYb8	288	47	N	N	AS	E	AGAATTACCCGCCAAG	TTCT/AT	
41	Alu	*FGFR2*	10	Bochukova et al. 2009 [288]	Apert syndrome	AluYk13	214	12	Y/5′TR	N	AS	E	AAAAGTTACATTCCG	TTTT/GA	
42	Alu	*FAS*	10	Tighe et al. 2002 [289]	ALPS	AluYa5	281	33	N	N	AS	I	AGAATATTCTAAATGTG	TTCT/AA	
43	Alu	*SERPING1*	11	Stoppa-Lyonnet et al. 1990 [290]	HAE	AluYc1	285	42	N	N	S	I	AAAAATACAAAAATTAG	TTTT/AG	
44	Alu	*HMBS*	11	Mustajoki et al. 1999 [291]	AIP	AluYa5	279	39	N	N	AS	E	AAGAATCTTGTCCC	TCTT/GA	
45	Alu	*ATM*	11	Qian et al. 2015 [158]	Hereditary Cancer	N/A	N/A	N/A	N/A	N/A	N/A	E	N/A	N/A	Pan-cancer panel testing
46	Alu	*GNPTAB*	12	Tappino et al. 2008 [292]	ML II	AluYa5	279	17	N	N	AS	E	AAAAACAACAACTGAG	TTTT/GA	
47	Alu	*BRCA2*	13	Miki et al. 1996 [293]	Breast Cancer	AluYc1	281	62	N	N	S	E	AATCACAGGC	GATT/AT	
48	Alu	*BRCA2*	13	Teugels et al. 2005 [294]	Breast Cancer	AluYa5	285	N/A	N	N	S	E	AAGAATCTGAACAT	TTCT/GC	3′ Processing 2 nt (5′-CT-3′)
49	Alu	*BRCA2*	13	Qian et al. 2015 [158]	Hereditary Cancer	N/A	N/A	N/A	N/A	N/A	N/A	E	N/A	N/A	Pan-cancer panel testing
50	Alu	*BRCA2*	13	Qian et al. 2015 [158]	Hereditary Cancer	N/A	N/A	N/A	N/A	N/A	N/A	E	N/A	N/A	Pan-cancer panel testing
51	Alu	*BRCA2*	13	Qian et al. 2015 [158]	Hereditary Cancer	N/A	N/A	N/A	N/A	N/A	N/A	E	N/A	N/A	Pan-cancer panel testing
52	Alu	*BRCA2*	13	Qian et al. 2015 [158]	Hereditary Cancer	N/A	N/A	N/A	N/A	N/A	N/A	E	N/A	N/A	Pan-cancer panel testing
53	Alu	*BRCA2*	13	Qian et al. 2015 [158]	Hereditary Cancer	N/A	N/A	N/A	N/A	N/A	N/A	E	N/A	N/A	Pan-cancer panel testing
54	Alu	*BRCA2*	13	Qian et al. 2015 [158]	Hereditary Cancer	N/A	N/A	N/A	N/A	N/A	N/A	E	N/A	N/A	Pan-cancer panel testing
55	Alu	*BRCA2*	13	Qian et al. 2015 [158]	Hereditary Cancer	N/A	N/A	N/A	N/A	N/A	N/A	E	N/A	N/A	Pan-cancer panel testing
56	Alu	*BRCA2*	13	Qian et al. 2015 [158]	Hereditary Cancer	N/A	N/A	N/A	N/A	N/A	N/A	E	N/A	N/A	Pan-cancer panel testing
57	Alu	*PMM2*	16	Schollen et al. 2007 [295]	CDG-Ia	AluYb8	263	10	Y/5′TR	N	AS	28 kb Deletion	No TSD	TTTT/AA	
58	Alu	*PALB2*	16	Qian et al. 2015 [158]	Hereditary Cancer	N/A	N/A	N/A	N/A	N/A	N/A	E	N/A	N/A	Pan-cancer panel testing
59	Alu	*BRCA1*	17	Peixoto et al. 2013 [161]	Breast and Ovarian Cancer Family	AluYc	191	60	Y/5′TR	N	AS	23.3 kb Deletion	No TSD	CTTT/AG	
60	Alu	*BRCA1*	17	Teugels et al. 2005 [294]	Breast Cancer	AluS	286	N/A	N	N	S	E	GAAAAAGAATCTGCTTT	TTTC/GA	
61	Alu	*BRCA1*	17	Qian et al. 2015 [158]	Hereditary Cancer	N/A	N/A	N/A	N/A	N/A	N/A	E	N/A	N/A	Pan-cancer panel testing
62	Alu	*NF1*	17	Wallace et al. 1991 [23]	NF1	AluYa5	282	40	N	N	AS	I/Splicing	AAAAAAAAAAACAT	TTTT/AA	First report of de novo Alu insertion
63	Alu	*NF1*	17	Wimmer et al. 2011 [296]	NF1	AluY	280	N/A	N	N	S	I	AAAAAATTCAG	TTTT/AA	Same insertion site as Wimmer et al.^a^
64	Alu	*NF1*	17	Wimmer et al. 2011 [296]	NF1	AluY	281	N/A	N/A	N	AS	I	N/A		
65	Alu	*NF1*	17	Wimmer et al. 2011 [296]	NF1	AluYa5	282	60	N	N	S	E	ATAAATAGCCTGGA	TTAT/AA	
66	Alu	*NF1*	17	Wimmer et al. 2011 [296]	NF1	AluYa5	284	120	N	N	AS	E	AAAAAACTTGCT	TTTT/GA	Same insertion site as Wimmer et al.^c^
67	Alu	*NF1*	17	Wimmer et al. 2011 [296]	NF1	AluYa5	281	N/A	N	N	AS	E	AAAAAACTTGCTGATGG	TTTT/GA	Same insertion site as Wimmer et al.^c^
68	Alu	*NF1*	17	Wimmer et al. 2011 [296]	NF1	AluYa5	284	110	N	N	AS	E	AATAAAACCTAAAGA	TATT/GA	
69	Alu	*NF1*	17	Wimmer et al. 2011 [296]	NF1	AluYa5	279	N/A	N	N	S	E	AAAAGAAGAACATAT	TTTT/GT	Same insertion site as Wimmer et al.^b^
70	Alu	*NF1*	17	Wimmer et al. 2011 [296]	NF1	AluYa5	264	60-85	Y/5′TR	N	AS	E	AAGAAGTGCGGTACCT	TCTT/GA	
71	Alu	*NF1*	17	Wimmer et al. 2011 [296]	NF1	AluYb8	249	121	Y/5′TR	N	S	E	AAAGCAGTGC	CTTT/AT	
72	Alu	*NF1*	17	Wimmer et al. 2011 [296]	NF1	AluYb8	288	N/A	N	N	AS	I	AAAAAAGAGAAAGACAA	TTTT/AA	Same insertion site as Wimmer et al.^a^
73	Alu	*NF1*	17	Wimmer et al. 2011 [296]	NF1	AluYb8	289	120	N	N	AS	E	AACAATGGTCTT	TGTT/AA	
74	Alu	*NF1*	17	Wimmer et al. 2011 [296]	NF1	AluYb8	288	78-178	N	N	S	E	AAACAATGATGTTA	TTTC/AA	3′ Processing of 1 nt (C)
75	Alu	*NF1*	17	Wimmer et al. 2011 [296]	NF1	AluYb8	288	118	N	N	S	E	AAAAGAAGAACATAT	TTTT/GT	Same insertion site as Wimmer et al.^b^
76	Alu	*NF1*	17	Wimmer et al. 2011 [296]	NF1	AluYb8	268	121	Y/5′TR	N	AS	I	AAAAAACAAACAAACA	TTTT/GT	
77	L1	*CYBB*	X	Meischl et al. 1998 [297], Brouha et al. 2002 [181]	CGD	L1 Ta	1722	101	Y/5′TR	Y (280)	S	E	AA	TGTT/GA	Maternal Meiosis I
78	L1	*CYBB*	X	Meischl et al. 2000 [298]	CGD	L1 Ta	836	69	Y/5′TR/INV	N	S	I/Splicing	AGAAATAACTATTTAA	TTCT/AA	
79	L1	*CHM*	X	van den Hurk et al. 2003 [177]	Choroideremia	L1 Ta	6017	71	FL	Y (119/406)	AS	E	AGAAGATCAATTAG	TTCT/AA	Insertion in Early Development
80	L1	*DMD*	X	Musova et al. 2006 [299]	DMD	L1 Ta	452	41	Y/5′TR/INV	N	AS	E	AAATATCTTTATATCA	ATTT/AA	
81	L1	*DMD*	X	Narita et al. 1993 [164]	DMD	L1 Ta	608	16	Y/5′TR	N	AS	E	No TSD	TCTT/AA	2 nt deletion
82	L1	*DMD*	X	Holmes et al. 1994 [176]	DMD	L1 Ta	1400	38	Y/5′TR/INV	Y(489)	S	E	AAATCATCTGCTGCT	ATTT/AA	First Report of L1 3′TR
83	L1	*DMD*	X	Yoshida et al. 1998 [300]	XLDCM	L1 Ta	530	73	Y/5′TR	N	AS	5′-UTR/Loss of mRNA	AAAAAAAACCTGGTAAA	TTTT/AT	Tissue specific loss of mRNA
84	L1	*DMD*	X	E Bakker & G van Omenn, personal communication	DMD	N/A	878	N/A	Y/5′TR	N	S	N/A	N/A	N/A	
85	L1	*DMD*	X	Awano et al. 2010 [301], Solyom et al. 2011 [302]	DMD	L1 Ta	212	118	Y/5′TR	Y (212)	AS	E	GAA	TTTC/AA	Orphan 3′-transduction
86	L1	*FVIII*	X	Kazazian et al. 1988 [22]	Hemophilia A	L1 Ta	3800	54	Y/5′TR	N	S	E	AAAGACAAACAAAAC	CTTT/AA	First report of de novo L1 insertion
87	L1	*FVIII*	X	Kazazian et al. 1988 [22]	Hemophilia A	L1 preTa	2300	77	Y/5′TR/INV	N	AS	E	AATGTTTCCTTCTTTTC	CATT/AA	
88	L1	*FIX*	X	Li et al. 2001 [266]	Hemophilia B	L1 Ta	463	68	Y/5′TR	N	S	E	AAAAATAGTGCTGATA	TTTT/AC	
89	L1	*FIX*	X	Mukherjee et al. 2004 [303]	Hemophilia B	L1 Ta	163	125	Y/5′TR	N	S	E	GAAAAATGGATTGT	TTTC/AT	
90	L1	*RP2*	X	Schwahn et al. 1998 [304]	XLRP	L1 Ta	6000	64	FL	N	S	I/Loss of mRNA	AAGACTGTAAGGTG	TCTT/AA	Interrupted polyA
91	L1	*RPS6KA3*	X	Martinez-Garay et al. 2003 [305]	Coffin-Lowry syndrome	L1 Hs	2800	Yes	Y/5′TR/INV	N	AS	E	AAGAAAACCTGCATTT	TCTT/AG	
92	L1	*ABDH5*	3	Samuelov et al. 2011 [306], Eli Sprecher, personal communication	CDS	N/A	FL	N/A	N	N/A	N	I/Splicing	N/A	N/A	
93	L1	*MLH1*	3	Qian et al. 2015 [158]	Hereditary Cancer	N/A	N/A	N/A	N/A	N/A	N/A	E	N/A	N/A	Pan-cancer panel testing
94	L1	*MLH1*	3	Qian et al. 2015 [158]	Hereditary Cancer	N/A	N/A	N/A	N/A	N/A	N/A	E	N/A	N/A	Pan-cancer panel testing
95	L1	*APC*	5	Miki et al. 1992 [200]	Colon cancer	L1Ta	520	222	Y/5′TR/INV	N	S	E	AAGAATAATG	TCTT/AA	Somatic Insertion/same insertion site as Halling et al. Alu
96	L1	*EYA1*	8	Morisada et al. 2010 [247]	BOR syndrome	L1 Hs	3756	None	Y/3′TR	N	AS	17 kb Deletion	No TSD	TCTC/AG	Internal Priming
97	L1	*FKTN*	9	Kondo-Iida et al. 1999 [307]	FCMD	L1Ta	1200	59	Y/5′TR	N	S	I/Splicing/6 nt Deletion	No TSD	TTTT/AA	
98	L1	*SETX*	9	Bernard et al. 2009 [308], Christine Zühlke, personal communication	AOA2	L1 Hs	1300	42	Y/5′TR/INV	N	S	E	GGAAGAATGTGAACTGGCTA	TTCC/AG	3′-processing 2 nt (5′-CC-3′)
99	L1	*PTEN*	10	Helman et al. 2014 [201]	endometrial carcinoma	L1 Hs	90	22	Y/5′TR	N	S	E	AAAGAATCATCTGGATTATAG	CTTT/AA	Somatic Insertion
100	L1	*HBB*	11	Divoky et al. 1996 [309]	β-thalassemia	L1 Ta	6000	107	FL	N	AS	I	AAAATAAAAGCAGA	TTTT/AT	
101	L1	*PDHX*	11	Mine et al. 2007 [310]	PDHc deficiency	L1 Hs	6086	67	FL	N	S	46 kb Deletion	No TSD	TTTT/AT	
102	L1	*SLCO1B3*	12	Kagawa et al. 2015 [157]	Rotor syndrome	L1 Ta-1d	5989	100	Near FL	N	S	I/Splicing	AAGAATTAATAGTGACAGT	TCTT/AC	0.054 Japanese Allele Frequency, may be “Hot L1”
103	L1	*RB1*	13	Rodriguez-Martin et al.2016 [202]	Familial Retinoblastoma	L1 Ta-1d	6044	33	FL	N	S	I/Splicing	AAATTATCTGTTTC	ATTT/AA	N/A
104	L1	*NF1*	17	Wimmer et al. 2011 [296]	NF1	L1 preTa	1800	N/A	Y/5′TR	N	S	E	AAAAACGAAACTGTGT	TTTT/AT	Untemplated 3′- T?
105	L1	*NF1*	17	Wimmer et al. 2011 [296]	NF1	L1 Ta	6000	N/A	FL	N	S	E	AAAAATCGAGGG	TTTT/AA	Untemplated 3′- T?
106	L1	*NF1*	17	Wimmer et al. 2011 [296]	NF1	N/A	2200	N/A	Y/5′TR/INV	N	AS	I/Splicing	AAGAAAATGGT	TCTT/AA	
107	SVA	*BTK*	X	Rohrer et al. 1999 [311], Conley et al. 2005 [258]	XLA	N/A	251	92	Y/5′TR	N	S	E	AGAAATGTATGAGTAA	TTCT/AT	Same insertion site as Conley et. al. Alu
108	SVA	*TAF1*	X	Makino et al. 2007 [312]	XDP	F	2627	62	FL	N	AS	I	AAAAAAAAAAAATGAAATAG	TCCT/AT	3′-Processing 3 nt (5′-CCT-3′)
109	SVA	*FIX*	X	Nakamura et.al. 2015 [156]	Hemophilia B	F	2524	28	FL	N	AS	E	AAATGGCACTAGAA	TTCC/AT	3′-Processing 1 nt (5′-C-3′)
110	SVA	*LDRAP1*	1	Wilund et al. 2002 [313]	ARH	E	2600	57	FL	N	S	I/Splicing	GAAACCTGTTTTCTC	TTTC/AA	
111	SVA	*SPTA1*	1	Hassoun et al. 1994 [314], Ostertag et al. 2003 [24]	HE and HPP	E	632	50	Y/5′TR/INV	Y (183/599)	S	E	GAAATTTGAAGACTTCCAAGT	TTTC/AA	Orphan 3′-transduction
112	SVA	*CASP8*	2	Stacey et al. 2016 [203]	Breast Cancer Susceptibility	E	2782	N/A	FL	N	AS	I/Decreased RNA	AAGAATTTGA	TCTT/AT	Protective against prostate cancer; active locus?
113	SVA	*A4GNT*	3	Nazaryan et al. 2015 [155]	Chromothripsis	E	502	None	Y/5′TR (VNTR)	N	AS	I	N/A	TTTT/GA	First report of large scale rearrangement and an insertion. Implicates retrotransposition in germline chromothripsis.
114	SVA	*HLA-A*	6	Takasu et al. 2007 [315]	Leukemia	F1	2000	45	FL	N/A	AS	14 kb Deletion	N/A	CCTT/AG	Novel SVA subfamily (F1)
115	SVA	*PMS2*	7	van der Klift et al. 2012 [154]	Lynch syndrome	F	2200	64	Y/5′TR (VNTR)	N	S	I/Splicing	AAGAATGTGCCATGTGA	TCTT/AA	SVA exonization
116	SVA	*FKTN*	9	Kobayashi et al. 1998 [162]	FCMD	E	3023	32	FL	N	S	3′UTR/Splicing	AAGAAAAAAAAAATTGT	TCTT/AA	
117	SVA	*PNPLA2*	11	Akman et al. 2010 [316]	NLSDM	E	1800	44	Y/5′TR	N	S	E	AAAGAGGCCCGG	CTTT/AG	
118	SVA	*SUZ1P*	17	Vogt et al. 2014 [153]	NF1	F1	1700	23	Y/5′TR (VNTR)	Y (282/160)	AS	I/Deletion of NF1	N/A	TTTT/AC	Largest reported insertion associated deletion (~1 Mb), somatic
119	SVA	*SUZ1P*	17	Vogt et al. 2014 [153]	NF1	F	1300	40	Y/5′TR (VNTR)	N	AS	I/Deletion of NF1	N/A	CTTT/AC	867 kb deletion, somatic
120	Processed Pseudogene	*CYBB*	X	de Boer et al. 2014 [152]	CGD	N/A	5739	100	FL	No	AS	I/Splicing	AAAACTCAAAGACTC	TTTT/AA	First reported de novo processed pseudogene (TMF1)
121	pA	*COL4A6*	X	Segal et al. 1999 [317]	Alport syndrome	N/A	N/A	70	N/A	N/A	AS	13.4 kb Deletion	No TSD	TTCT/AT	
122	pA	*AGA*	4	Jalanko et al. 1995 [318]	AGU	N/A	N/A	37	N/A	N/A	AS	2 kb Deletion	No TSD	TTCT/AA	
123	pA	*BRCA2*	13	Wang et al. 2001 [319]	Breast Cancer	N/A	N/A	35	N/A	N/A	S	6.2 kb Deletion	No TSD	TTCT/AA	
124	pA	*NF1*	17	Wimmer et al. 2011 [296]	NF1	N/A	130	120	N/A	N/A	AS	E	AAGAAA	TCTTNAA	

Data for this table were compiled from the primary references listed and reports prior to 2009 are reviewed in the following: Ostertag and Kazazian 2001 [35], Chen et al. 2006 [150], Belancio et al. 2008 [151], Hancks and Kazazian 2012 [86]

A few insertions were left off the list as they were common polymorphisms or did not cause disease. The following websites and databases were used in the analysis: http://www.repeatmasker.org/, Repbase (http://www.girinst.org/), http://dbrip.brocku.ca/, The following symbols, ^a^,^b^,^c^, indicate same insertion site in Wimmer et al. [296]

*Abbreviations: TR* truncation, *INV* inversion, *E* exon, *FL* full-length, *I* intron

*Disease acronyms: ADOA* Autosomal dominant optic atrophy, *AGU* Aspartylglucosaminuria, *AIP* Acute intermittent porphyria, *ALD* Adrenoleukodystrophy, *ALPS* Autoimmune lymphoproliferative syndrome, *AOA2* Ataxia with oculomotor apraxia 2, *ARH* Autosomal recessive hypercholesterolemia, *BOR* Branchio-oto-renal syndrome, *CDG-Ia* Congenital disorders of glycosylation type Ia, *CDS* Chanarin-Dorfman syndrome, *CGD* Chronic granulomatous disease, *DMD* Duchenne muscular dystrophy, *FAP* Familial adenomatous polyposis, *FCMD* Fukuyama-type congenital muscular dystrophy, *FHH and NSHPT* Familial hypocalciuric hypercalcemia and neonatal severe hyperparathyroidism, *GKD* Glycerol kinase deficiency, *HAE* Hereditary form of angioedema, *HE and HPP* Hereditary elliptocytosis and hereditary pyropoikilocytosis, *HIGM* Hyper-immunoglobulin M syndrome, *HNPCC* Hereditary non-polyposis colorectal cancer syndrome, *LPL* Lipoprotein lipase, *MLII* Mucolipidosis Type II, *MWS* Mowat-Wilson syndrome, *NF1* Neurofibromatosis Type I, *PDHc* Pyruvate dehydrogenase complex deficiency, *NLSDM* Neutral lipid storage disease with subclinical myopathy, *RP* Retinitis pigmentosa, *Type 1 ATP* Type 1 antithrombin deficiency, *XDP* X-linked dystonia-parkinsonism, *XLA* X-linked agammaglobulinemia, *XLDCM* X-linked dilated cardiomyopathy, *XLRP* X-linked retinitis pigmentosa, *XSCID* X-linked severe combined immunodeficiency

The cleavage of the DNA bottom-strand liberates a 3′-OH which will serve as the primer used by ORF2p for reverse-transcription. It is postulated that the T-rich bottom-strand basepairs with the LINE-1 RNA polyA tail and perhaps in some instances, a nuclease activity associated with the LINE-1 RNP processes the 3′-bottom strand to obtain a better primer. 3′-processing can be observed biochemically and for genomic insertions where the cleavage site appears to be absent by annotation (e.g. YYYY/YR), the actual site is merely obscured by this activity [85, 86].

Following bottom-strand cleavage, ORF2p initiates reverse-transcription of the LINE-1 RNA to generate the first strand of LINE-1 cDNA [68, 78]. Cleavage of the DNA top-strand seems to occur following the bottom-strand nick in a stepwise manner after initiation of first-strand cDNA synthesis [69]. That said, sequence features in some LINE-1 insertions, namely 5′-inversions and target-site deletions, suggest that top-strand cleavage may occur prior to completion of bottom-strand cDNA synthesis. While sequence-specificity for top-strand cleavage has yet to be defined, sequence distance likely plays a role as the majority of target-site duplications are within 4-20 bp in length [6, 37, 84, 86]. A potential suspect for top-strand cleavage could be the additional nuclease activity observed in vitro in LINE-1 RNPs [78, 85]. Next, top-strand cDNA synthesis ensues probably by ORF2p which displays DNA-dependent DNA synthesis activity in vitro [87].

In contrast to DNA transposon and ERV insertions, most LINE-1 insertions are not a full 6 kbp in length (Fig. 2b). The majority of genomic LINE-1 s (>99 %) are grossly truncated at their 5′-end (Fig.2c) or contain a 5′-inversion (Fig. 2e) of the LINE-1 sequence [37, 88]. Although ~ one-third of the human-specific LINE-1 s are full-length, indicating most full-length elements have been selected against throughout primate evolution [89] and even recently since the human-chimpanzee divergence [90], some LINE-1-containing loci display signatures of positive selection [91]. The lack of LINE-1 RT processivity during cDNA synthesis is unlikely to contribute to short insertions as non-LTR RTs, including ORF2p, are highly processive in vitro [87, 92]. Currently, it is speculated that conflict with host factors, that serve as defenders of the genome against LINE-1 parasites [93, 94] such as apolipoprotein B mRNA editing enzyme catalytic subunit 3A (APOBEC3A) or DNA repair factors [44, 45, 95] like ataxia telangiectasia mutated (ATM), limit the size of a LINE-1 insertion [95]. Ongoing studies will determine whether this conflict interferes with cDNA synthesis resulting in a shortened first-strand cDNA or whether some unknown factor attacks and perhaps cleaves a full-length first-strand cDNA basepaired with the LINE-1 RNA. Conversely, it has not escaped notice that LINE-1 sequences contain numerous sequence motifs resembling the LINE-1 EN cleavage site on what would be deemed the bottom-strand. Although no ribonuclease H (RNaseH) activity has been associated with LINE-1 proteins and perhaps it is counterintuitive, it may be possible that LINE-1 cleaves itself as part of a multifaceted molecular arms-race including but not limited to: 1) AT-rich codon optimization in the ORFs to limit DNA methylation which has the potential to alter coding via cytidine deamination, 2) low ORF2 protein expression to escape innate immunity, and 3) maintenance of only the minimum number of full-length insertions deployed throughout the genome in order to survive and to cloak itself from purifying selection. In contrast, a yet unidentified host-encoded nuclease, such as a factor distantly related to ORF2p with a preference for AT-rich motifs may in part explain LINE-1 5′-truncations. Overall, many of the key steps in LINE-1 retrotransposition have been defined; yet, gaps still exist in our understanding of this selfish gene’s lifecycle.

### *Trans*-mobilization of non-autonomous elements

#### Alu elements

In addition to mobilizing its own RNA, LINE-1 proteins retrotranspose a myriad of other RNAs. For instance, the most abundant retrotransposon in the human genome by copy number is the Short INterspersed Element (SINE) Alu [6]. Its name originates from human DNA renaturation studies which identified an abundant ~300 repetitive nucleotide sequence that contained the *Alu*I restriction endonuclease cleavage site [96]. Alu RNAs are primate-specific [97, 98] non-coding RNA Pol III transcripts [98] derived from the 7SL RNA [99, 100], a component of the signal recognition particle. While Alu elements contain their own transcriptional signals [101] (A and B box, and Pol III terminator (TTTT)) [102] and end in a polyA tail of varying length, transcriptional robustness is largely dictated by the presence of upstream enhancer elements [103, 104] (Fig. 1b) . The Alu polyA tail, which is part of the element, differs from the LINE-1 polyA, which is presumably added via the canonical polyadenylation pathway. Along with being transcribed via internal signals, these elements are frequently incorporated into the transcriptome via exonization [105]. An antisense Alu element contains certain sequence features that poise this SINE for splicing. In particular, a pyrimidine-rich tract is generated by the reverse complement of the polyA tail along with a CAG trinucleotide motif, which together generate a very strong splice acceptor motif.

The Alu’s evolutionary origins provide insight into how it has become the most abundant retrotransposon in the human genome. Namely, evolution from 7SL RNA [100] followed by monomer duplication [106–108], which increased SRP9/14 binding sites, coupled with increased protein levels of SRP 14 due to triplet repeat expansion seeded by a point mutation in the anthropoid ancestor [109], enhanced localization of this non-coding RNA to the ribosome where it can hijack the LINE-1 protein machinery [110]. Cell-culture retrotransposition assays and mutational analysis by Devine and colleagues have shown that Alus with less secondary structure similarity to 7SL have decreased LINE-1-mediated retrotransposition [111]. Structural analysis has revealed Alu in complex with the SRP 9/14 proteins [110, 112].

Following incorporation into the LINE-1 RNP, Alu integration likely follows in a fashion similar to LINE-1. Based on *trans*-mobilization cell culture assays, engineered Alu elements require a polyA tail and appear to only need transfected LINE-1 ORF2 for retrotransposition [71]. Subsequently, it was demonstrated that transfecting increasing amounts of an ORF1 plasmid enhances Alu retrotransposition [113]. One might infer from these data that endogenous ORF1 and ORF2 generated from distinct LINE-1 elements could serve to retrotranspose Alus.

In contrast to LINE-1, most Alus are full-length. However, 5′-truncated Alu elements have been identified in human genomes [114] and as *de novo* insertions resulting in disease (Table 1). Over evolutionary time, Alus appear to be more tolerated than LINE-1 in introns [115], which may be due to their decreased effectiveness over evolutionary time in mediating ectopic homologous recombination when compared to LINE-1. Alus are also commonly found in 3′-UTRs where they may serve as small RNA binding sites [116, 117] or serve as substrates for the RNA editing enzyme adenosine deaminase acting on RNA (ADAR) when at least two inverted Alus are present [118–120]. Also, base-pairing between Alus embedded in mRNA 3′-UTRs and long non-coding RNAs can be involved in directing Staufen-mediated RNA decay [121].

#### SVA elements

The youngest active human retrotransposon is named after the sum of its parts SINE-VNTR-Alu (SVA). SVA elements are ~2 kb hominid-specific non-coding composite elements [24, 122, 123]. The structure of an SVA (Fig. 1c) [124–126], starting from its 5′-end, is 1) a CCCTCT mostly pure repeat ranging from a few copies up to a hundred (also known as the hexamer), 2) an Alu-like domain derived from two Alu antisense fragments, 3) a variable number of very GC-rich tandem repeats (VNTR), 4) a SINE-R domain sharing sequence homology to the *env* gene and right LTR from a HERV-K, and 5) a polyA tail of varying length similar to LINE-1. From element to element within the human genome, these sequences display more structural sequence variation than LINE-1 and Alu [125–127], primarily because of changes in hexamer and VNTR copy number along with 5′- [127, 128] and 3′- transductions [24, 65]. There are approximately 2700 SVA elements in the human genome reference sequence [123] which differs dramatically from LINE-1 and Alu copy number, ~500,000 and ~1,000,000 copies, respectively. Due to its more recent discovery relative to LINE-1 and Alu elements, less is known about SVA biology.

The nature of the SVA transcriptional unit or SVA promoter has not been completely resolved, but SVAs are presumed to be Pol II transcripts due to the poly A tail downstream of a canonical polyA signal (AATAAA). Furthermore, untemplated guanosines [127, 129, 130] have been identified at the 5′-end of full-length insertions, similar to LINE-1, which likely represent reverse-transcription of the 7mG cap. Initiation of SVA transcription can be broadly grouped into 4 classes [126–128]: 1) transcription initiation from within the hexamer, 2) transcription initiation downstream of the hexamer, 3) transcriptional initiation 5′- of the SVA, which can lead to retrotransposition of upstream sequences (e.g. 5′-transduction) (Fig. 2f), and 4) transcription initiation in an upstream exon followed by splicing into SVA which results in a chimeric transcript (Fig. 2h).

It is currently unclear how or where SVA RNA interacts with the LINE-1 proteins. It has been predicted that perhaps the SVA RNA is localized to the ribosome [24, 131] via base-pairing interactions between the SVA Alu-like domain and Alu RNAs. This may be the case for some SVAs, however the recent discovery of a human-specific SVA subfamily generated via splicing from the first exon of the microtubule associated serine/threonine kinase 2 (*MAST2*) gene into the 3′-end of the Alu-like domain [127, 128, 132], suggests that basepairing with Alu at the ribosome is not a requirement. SVAs require ORF2p for retrotransposition [24, 129, 133], whereas the requirement for ORF1p is less clear, in part, because the contribution of endogenous ORF1p for engineered SVA retrotransposition is unknown. Cell-culture retrotransposition assays and deletion analysis indicate that SVAs require the 5′-end (hexamer and Alu-like domain) of the element to retrotranspose [130, 133]. Additional requirements for LINE-1-mediated retrotransposition are currently being investigated using a comparative strategy involving cell culture assays [134, 135] with the newly identified composite VNTR elements like LAVA in gibbons [136–138].

Once an SVA RNA is incorporated into the RNP, integration is hypothesized to occur in a similar manner to LINE-1. SVA insertions share many similarities to LINE-1. Other than typical LINE-1 hallmarks (target-site duplication, insertion at LINE-1 EN site, end in polyA tail), both LINE-1 and SVA insertions occasionally contain 3′-transductions [24, 62, 65, 139]. Some SVA insertions differ from classical LINE-1 insertions by containing 5′-transductions, which are almost non-existent for LINE-1 [6, 82] yet ~10 % of all SVAs contain transduced sequence via upstream transcriptional initiation [127, 128]. Unlike LINE-1 s, SVAs are occasionally 3′-truncated due to premature polyadenylation mediated by polyA signals located in the SINE-R [127]. Another major difference between LINE-1 and SVA insertions, both in the genome [127] and those recovered from cell culture retrotransposition assays [129, 130, 133], is that most SVAs are full-length while less than 5 % of LINE-1 s recovered from cell culture assays are full-length [6, 84]. Indeed, full-length SVAs and LINE-1 do differ in length (SVAs range from ~1 kb to almost 5 kb and LINE-1 = 6 kb), yet many if not most LINE-1 genomic insertions are under 1.5 kb [6]. A notable difference between LINE-1 and SVA is the sequence composition; LINE-1 s are very AT-rich while SVAs are very GC-rich.

#### Retrotransposition of splicesomal RNAs

In addition to Alu and SVAs, other RNAs encoded by the genome can be retrotransposed. Many small splicesomal RNAs are commonly integrated by LINE-1 into genomes with U6 being the most frequent (Fig. 1e) [140–144]. Interestingly, U6 retrotransposition events are often characterized as chimeric insertions [140, 144]. Specifically most are fused with a LINE-1 or an Alu element at the 3′-end of the U6 sequence [144]. In addition to evidence from the genome, chimeric U6-LINE-1 insertions have been identified and investigated using cell culture retrotransposition assays [84, 144].

#### Retrotransposition of protein-coding transcripts

Along with small abundant RNAs, LINE-1 can mobilize protein-coding RNAs [145, 146]. Following retrotransposition, these insertions are referred to as processed pseudogenes (PPs) due to their lack of introns (Fig. 1d). PPs contains all of the hallmarks of LINE-1-mediated retrotransposition (target-site duplications, 5′-truncations, 5′-end inversions, end in a polyA tail of variable length). The reference human genome sequence contains approximately ~8000 processed pseudogenes [147] with the most abundant being sequences encoding ribosomal protein RNAs [148]. Retrotransposition is thought usually to inactivate PPs due to the loss of regulatory elements such as promoter sequences.

## Mechanisms by which retrotransposons can cause disease

Retrotransposons can potentially cause disease by a variety of mechanisms [149]. Most of the 124 disease-causing insertions [35, 86, 150–161] reported to date inactivate gene function through insertional mutagenesis or aberrant splicing. Indeed, the precise mechanism of gene inactivation may be more detailed. For example, a LINE-1 insertion into an exon or an intronic insertion that is spliced into may result in a frameshift mutation that will result in nonsense-mediated RNA decay. In contrast, depending on the site of insertion, the retrotransposon may result in an alternative C-terminus of a protein which can in turn alter function of the gene leading to disease. This is best exemplified by the SVA element insertion in the fukutin (*FKTN*) gene which causes fukuyama muscular dystrophy [162, 163]. Here, alternative splicing of the *FKTN* mRNA into the SVA located in the 3′-UTR generates a protein that is mislocalized from the Golgi to the endoplasmic reticulum [163].

Another major mechanism by which LINE-1-mediated insertions result in disease is through target-site deletions (Fig. 2j) [82, 83]. Deletions associated with *de novo* LINE-1-mediated insertions range from a few basepairs [164] up to a megabase [153]. LINE-1 [165], Alu [166], and SVA [167] associated target-site deletions have also been identified in the human and primate genomes. Thus, these deletions in the short-term may result in disease but may serve as a means by which retrotransposons contribute to genome evolution. Notably, retrotransposon sequences can also generate genetic deletions via non-allelic homologous recombination (NAHR) [168] which is independent of TPRT and DNA breakage mediated by LINE-1 ORF2p. NAHR is most frequently observed for Alu elements presumably due to their high copy-number and results in structural variation which can lead to genetic disease. These deletions may be generated via mispairing of two retrotransposon sequences on the same strand usually on homologous chromosomes, while crossing over between two retrotransposon sequences inverted relative to each other may result in an inversion [149, 169].

Additional hypothesized mechanisms by which new LINE-1, Alu, and SVA element insertions may disrupt gene function relate to epigenetic changes at the site of integration. All three elements are known to be methylated at CpGs. LINE-1 [170, 171] and SVA DNA [172], in the 5′-UTR and VNTR, respectively, are known to be densely methylated in somatic tissue. Interestingly, SVAs were initially identified by one group when carrying out a restriction endonuclease based assay to identify methylated sequences in the human genome [172]. In this study, SVA comprised >70 % of one of the libraries of methylated sequence. Along the epigenetic spectrum, alterations in local histone modifications following LINE-1 insertion have been described in teratocarcinoma cell lines [173]. Specifically, recruitment of a histone-deacetylase enzyme by some unknown mechanism or signal to LINE-1-target sites results in deacetlyation of histone tails. Similarly, a new study reports that Sirtuin-6 (SIRT6) can repress LINE-1 [174] by binding the 5′-UTR and ribosylating KRAB-associated protein-1 (KAP1), a major corepressor. This posttranslational modification is important for KAP1 to interact with heterochromatin protein-1α (HP1α). Interestingly, over time (e.g. ageing) SIRT6 is depleted at LINE-1 loci. Although no specific examples have been reported thus far for disease-causing insertions, experimental evidence indicates epigenetic silencing following LINE-1, Alu, or SVA insertion in a gene may result in reduced mRNA expression from a given gene. In contrast, loss of epigenetic mediated repression may lead not only to expression of retrotransposons but also neighboring genes. For example, one report demonstrated that loss of DNA methylation occurs at an intronic LINE-1 insertion near the hepatocyte growth factor receptor (*MET*) gene which leads to expression of a LINE-1-MET fusion transcript encoding a truncated form of this protein known to be oncogenic [175].

### New reports of LINE-1-mediated insertions causing Mendelian disorders

Since our last survey of disease-causing insertions [86], 28 more have been reported in the literature. Disease-causing insertions have been priceless in regards to our understanding of human retrotransposon biology. Genetic disease phenotypes serve as markers to identify *de novo* retrotransposition events. It has been almost 30 years since the first *de novo* retrotransposon insertion was identified in the factor VIII (*F8*) gene of a Hemophilia A patient by Kazazian and colleagues [22]. LINE-1-mediated insertions have been associated with autosomal dominant, autosomal recessive, and X-linked genetic disorders (Table 1). Disease-causing insertions have aided in the recovery of active retrotransposons used in cell-culture retrotransposition assays [41]. Furthermore, these insertions have confirmed and revealed phenomena, such as 3′-transductions [176], observed in cell culture and genomic studies.

Neurofibromatosis Type I is an autosomal dominant disorder caused by mutations in the *NF1* gene. Recently, while characterizing genetic deletions in the neurofibromin 1 (*NF1*) gene, an SVA insertion associated with a 867 kb deletion in one individual and an SVA in a different individual associated with a 1 MB deletion were found [153]. These two insertions represent the largest genomic deletions caused by a *de novo* insertion to date. Using sequence analysis, the authors were also able to identify the source elements for both insertions. One insertion was generated from a full-length SVA located on chromosome 6 belonging to the human-specific subfamily F. The other insertion was generated from an element on chromosome 10 belonging to the human specific SVA_F1 (*MAST2*) subfamily. The element on chromosome 10 has been associated with other SVA disease-causing insertions and is thought to be the source element for at least 13 genomic SVAs [127, 128]. Interestingly, both insertions were somatic. One patient had the SVA-associated deletion in 93 % of her blood cells (absent in 7 % of her blood cells); while the grandmother of the other patient who passed on the insertion had the SVA in 75 % of her blood cells (absent in 25 % of the blood cells).

Somatic mosaicism has been described for disease-causing insertions as in a LINE-1 retrotransposition event into the choroideremia (rab escort protein 1) (*CHM*) gene [177]. More and more evidence is accumulating that somatic insertions may be more common than previously appreciated and perhaps the norm [178–180]. In addition to disease-causing insertions, insights into somatic mosaicism generated by LINE-1 activity were first gained from two mouse studies: one investigating retrotransposition of engineered LINE-1 s in the brain [180] and the other studying LINE-1 inheritance [178]. Next-generation sequencing of cancer genomes and brain samples is starting to uncover a wealth of somatic insertions. The current thought in the field is that many, if not most, LINE-1-mediated insertions are not inherited despite what had been previously inferred based on the abundance of genomic insertions.

Most *de novo* retrotransposition events are likely benign, however coupled with a loss-of-function mutation on the other allele the insertion may result in recessive genetic disease (e.g. compound heterozygosity). A study analyzing the genetic basis for Rotor syndrome [157], an autosomal recessive disorder, uncovered patients homozygous for a near full-length LINE-1 insertion (lacking 24 nt from the 5′-end relative to LINE-1.3) in intron 5 of solute carrier organic anion transporter family member 1B3 (*SLCO1B3*). This insertion results in aberrant mRNA splicing and ultimately loss of SLCO1B3 protein expression in liver tissues. As Rotor syndrome is a digenic disorder the homozygous LINE-1 insertion alone is not sufficient to cause Rotor syndrome; these patients are also homozygous for a nonsense mutation in the downstream solute carrier organic anion transporter family member 1B1 (*SLCO1B1*) gene. LINE-1_*SLCO1B3*_ may represent a population-specific “hot LINE-1” with a gene frequency of ~6 % in Japanese individuals. LINE-1_*SLCO1B3*_ contains intact reading frames with ORF1 being 100 % identical to the LINE-1 amino acid consensus and LINE-1_*SLCO1B3*_ ORF2 containing three amino acid changes relative to the consensus LINE-1 sequence. A LINE-1 whose sequence is close to the amino acid consensus is typically very active in cell culture retrotransposition assays. For, example LINE-1_*LRE3*_ [181], which is one of the most active LINE-1 s isolated to date shares 100 % amino acid identity with the consensus sequence. In addition, a survey of highly active (“hot”) LINE-1 s, recently identified a very active element also lacking the first 21 nt (118 % of LINE-1.3) [40]. The first nucleotide of LINE-1_*SLCO1B3*_ –a guanine- may actually represent reverse-transcription of the 7mG cap, a phenomenon often observed for very active elements, as most full-length LINE-1 s have a thymine at this same position in the 5′-UTR. Although LINE-1_*SLCO1B3*_ may appear 5′-truncated, it is more probable that an alternative transcriptional start site may have been used at the source locus or that the source locus was lacking the first 24 nts. Other instances of LINE-1-mediated insertions associated with recessive diseases are typically due to a founder effect or consanguinity.

LINE-1-mediated retrotransposition events and inactive retrotransposons have long been considered to be agents of genome instability. A new study [155] analyzing a germline chromosome shattering event - a phenomenon referred to as chromothripsis [182, 183] - that can also occur in cancer, which consisted of 7 breakpoints and rearrangements between two chromosomes, identified a 502 bp 5′-truncated SVA element insertion spanning a breakpoint associated with a 110 kb deletion. This SVA element belongs to a young active subfamily and may be derived from a full-length SVA on chromosome 7. Additional analysis of genomic sequence prior to DNA shattering identified two Alu elements on the same strand flanking the 110 kb sequence that was deleted. Furthermore, two antisense Alus were identified at breakpoints junctions involving an inversion in this chromothripsis event. Unexpectedly, sequence motifs resembling LINE-1 endonuclease cleavage sites were identified at exact breakpoints. A model was developed that integrated Alu-mediated chromosome looping and LINE-1-mediated SVA retrotransposition to account for the genome configuration following chromothripsis [155].

To date, the DNA damage agents causing chromothripsis and the mechanisms driving the rearrangement of chromosomal fragments are poorly understood. [184–186]. Several hypotheses have been generated to explain chromothripsis [184–186] including: 1) replication fork collapse coupled with template-switching and 2) ionizing radiation followed by DNA repair via the non-homologous end-joining pathway. More recently, experimental analysis has demonstrated that partitioning of chromosomes into micronuclei can result in chromothripsis and may explain why chromothriptic rearrangements are restricted to a limited number of chromosomes [187]. In addition, another study has provided evidence that three prime repair exonuclease 1 (TREX1) may cause rearrangements reminiscent of chromothripsis between dicentric chromosomes formed by telomere fusion [188].

Although the new study [155] represents only one instance of active and inactive retrotransposons associated with chromothripsis, it is tempting to speculate that an endonuclease, such as ORF2p expressed in germ cells, in early development, and in some cancer cells may play a role. Finally, although a bit tenuous, it is worthwhile to note that “kataegis,” the localized hypermutation frequently observed near DNA breakpoint junctions in chromothripsis [188, 189] and cancers [190], is thought to be caused by the LINE-1 restriction factors [94, 191–194]- the APOBEC3 proteins [195]. APOBEC3A control of LINE-1 typically results in no observed remnants of LINE-1 sequence at the target-site due to uracil DNA glycosylase activity following APOBEC3A deamination of the TPRT intermediate [94]. Perhaps kataegis is a consequence of APOBEC3 defense of the genome against retrotransposons in cancers.

Contemporary LINE-1, Alu, and SVA activity has been documented, in part, due to new insertions causing genetic disease. A long-standing question in the field is whether processed pseudogene formation (e.g. retrotransposition of cellular mRNAs, retrogenes) is ongoing in humans? Despite the name, retrogenes may serve as a crucible for new genes. Retrogenes have the potential for subfunctionalization or neofunctionalization. For example, 5′-truncation or point mutations could in principle generate a negative regulator of the parent gene. One report noted that processed pseudogenes coupled with 5′-inversion may be a means to generate new genes with novel N-termini [196]. Likewise, it is interesting that many large DNA viruses, such as poxviruses, contain many genes that share homology to host genes, lack introns, and are flanked by A-rich sequences; perhaps, implicating LINE-1 activity in the evolution of viral genomes.

Analysis of the 1000 genomes sequencing and Cancer Genome Atlas data has identified 48 polymorphic processed pseudogenes [197], thus indicating retrotransposition of cellular mRNAs in recent human history. A new study demonstrated ongoing processed pseudogene formation when the investigators identified an insertion of a partly processed TMF1 gene transcript into the cytochrome b-245, beta polypeptide (*CYBB*) gene of a chronic granulomatous disease patient [152]. Uniquely, this insertion was very large (~5.8 kb) and represented an RNA that utilized a noncanoncial polyA signal [152]. The insertion was flanked by a target-site duplication, inserted at a LINE-1 EN cleavage site, and ended in a 3′- polyA tail. The authors also demonstrated that the mother of the patient displayed somatic mosaicism for the insertion consistent with retrotransposition in early development [152]. Along these lines, retrotransposition of an almost full-length centromere protein W (*CENPW*) RNA, lacking 7 bp relative to the annotated TSS, into exon 8 of Poc1 centriolar protein A (*Poc1a*) resulted in growth insufficiency and male infertility in mouse [198] (insertion size = 495 bp). This insertion displays all of the hallmarks of LINE-1-mediated retrotransposition (target-site duplication, insertion at EN cleavage site, and 3′-polyA tail).

### Other diseases

It is well-established that retrotransposition can occasionally result in human genetic disease. Of late there has been a great effort to determine whether these selfish genetic elements may contribute to complex diseases such as cancer, autoimmunity, and neuropsychiatric disorders.

#### LINE-1 s and cancer

Genomic instability is a hallmark of cancer [199]. Notably, one of the first disease-causing insertions reported was an LINE-1 insertion into the adenomatous polyposis coli (*APC*) gene of a colon cancer patient described by Nakamura and colleagues [200]. That insertion was somatic as it was absent in normal colon from the patient. Likewise, a very short somatic LINE-1 insertion (112 bp) was identified from exome data using TranspoSeq analysis in exon 6 of phosphatase and tensin homolog (*PTEN*) of an endometrial carcinoma [201]. Two new reports further indicate that cancer can be initiated by retrotransposition-mediated gene inactivation. The first example is a full-length LINE-1 insertion located in intron 14 of the tumor-suppressor retinoblastoma 1 (*RB1*) which results in retinoblastoma in the proband and his father [202]. The authors’ determined that this insertion was *de novo*, as it was absent from the father’s parents and the proband’s brother. The insertion causes aberrant RB1 splicing due to its precise integration into the splice acceptor site (target-site duplication (uppercase) tttt/AAATTATCTGTTT**C/ag**, splice acceptor trinucleotide motif in bold).

The second new report involves the use of population whole-genome sequencing to identify a full-length SVA_E_ insertion (2792 bp in length) into intron 8 of the caspase 8 (*CASP8*) gene associated with increased susceptibility to cutaneous basal cell carcinoma (BCC) and breast cancer [203]. It is thought that this SVA insertion accounts for the previously reported germline SNP in *CASP8* linked to BCC. The mechanism by which the antisense SVA insertion results in decreased CASP8 expression in breast cancer is unclear but it is not thought to be due to aberrant splicing. Extensive genotyping analysis indicated that the same SVA insertion into *CASP8* confers protection against prostate cancer in the same populations. This SVA locus has also been active in recent human history as evidenced by a full-length SVA insertion on chromosome 19 containing a 288 bp 3′-transduction derived from intron 8 *CASP8* sequence.

Although, LINE-1-mediated insertions have been identified in tumor suppressor genes, the overall absence of insertions in these genes has led researchers to focus on the contribution of LINE-1-mediated retrotransposition to cancer progression instead of cancer initiation. Numerous studies by independent labs over the past several years have reported extensive retrotransposition and/or LINE-1 protein expression in a variety of cancer types [201, 204–211]. Our recent studies [205, 207] demonstrate that LINE-1 insertions can occur in somatic gastrointestinal tissues, and that they can be carried forward essentially clonally in the cancers. We postulate that these somatic insertions contribute to the evolvability of cancer and its progression in the presence of limited resources and competition from not only the host but perhaps from other proximal competing cancer cells. Specifically, many somatic insertions may be benign, but following cancer initiation and the onslaught of other types of mutation including deletions, these insertions have the potential to optimize different cellular networks or if full-length seed new agents of adaptability during cancer progression.

A largely unexplored question is whether LINE-1 proteins play yet undefined roles in cancer [212]. For example, ORF2p may be a source of endonuclease activity contributing to additional genomic rearrangements in these already unstable cells. Furthermore, LINE-1 RT activity may be a means to mend DNA breaks similar to what has been observed for LINE-1 endonuclease independent insertions (Fig. 2k) [213–215]. Although completely speculative, based on LINE-1 endonuclease independent insertions at telomeres in cell culture [216], perhaps on occasion, LINE-1-mediated insertions may aid in telomere elongation in some cancers. In addition, ORF2p expression may have important roles in cancer onset and progression through perturbation of regulatory networks [217–220].

#### LINE-1 s and autoimmunity

Autoimmunity is characterized by the immune system attacking “self.” Some autoimmune disorders such as Aicairdes-Goutieres syndrome are caused by mutations in genes, such as *TREX1* or SAM domain and HD domain 1 (*SAMHD1*), known to inhibit LINE-1 activity [221–223]. More recently, it has been demonstrated that a pattern-recognition receptor (PRR) named cyclic GMP-AMP synthase (cGAS) serves as a sensor for cytoplasmic DNA and activates the interferon response in the absence of the DNase TREX1 [224]. Notably, cell culture and in vitro studies have shown that cGAS can activate the immune response not only by binding double-stranded DNA [225] but also by binding RNA: DNA hybrids [226]. Thus, cGAS or other PRRs may serve as critical cytoplasmic sentinels against retrotransposon replicative intermediates.

Autoantibodies are a hallmark of autoimmune disorders. Antibodies against the RNA binding protein Ro60 are detected in systemic lupus erythematosus and Sjorgen’s syndrome. Interestingly, Ro60 RNPs reactive to autoantibodies contained Alu RNAs [227]. Similarly, Alu RNAs have also been implicated in age-related macular degeneration. During disease progression, expression of the microRNA processing enzyme DICER is reduced in retinal pigmented epithelium (RPE) [228]. Surprisingly, knockdown of DICER in human and mouse RPE results in an increase in Alu or B1 and B2 SINE RNA [228]. Knockdown of Alu RNAs using antisense oligonucleotides halts RPE degeneration driven by DICER knockdown in primary RPE culture. It is thought that the loss of DICER and an increase in Alu RNA leads to NLRP3 inflammasome activation resulting in cell death via Caspase-1 activation [229]. Interestingly, nucleoside reverse-transcriptase inhibitors known to inhibit LINE-1 activity [230] can block RPE degeneration and inflammasome activation in mice injected sub-retinally with a plasmid expressing Alu [231].

The ability of retrotransposon replicative intermediates (e.g. RNA, cDNA) to trigger the innate immune response - activation of apoptotic pathways or interferon signaling- is consistent with a vital role for the immune system in protecting the cell and genome from TEs like LINE-1. It has been speculated that many key innate immunity factors such as APOBEC3 first evolved to control retrotransposition. Although several examples already exist, it is highly likely that additional immunity factors known to inhibit viral replication will be shown to also inhibit LINE-1 activity. In addition, while insertional mutatgenesis is thought to be the primary means by which retrotransposons result in human disease, these highlights from the literature indicate that the RNAs themselves may be toxic to host fitness. It remains to be determined whether individuals harboring diseases associated with an increase in retrotransposon RNA have an increase in endogenous LINE-1 mediated retrotransposition.

## LINE-1 s and neuronal diversity

A little over a decade ago, an interesting observation was made by Muotri, Gage, and colleagues regarding which cells are permissive for LINE-1 retrotransposition [180]. Prior to their work, most retrotransposition was thought to occur in the germline as evidenced by the ~500,000 LINE-1 copies in the human genome. Using engineered LINE-1 s, the authors detected LINE-1 retrotransposition in rat neuronal progenitor cells and in the brain of mice carrying an engineered LINE-1 marked with GFP [180]. These data demonstrated that engineered LINE-1 retrotransposition in the brain resulted in somatic mosaicism.

After a few years, the major question of whether endogenous LINE-1 was retrotransposing in the brain in vivo would be answered. Faulkner and colleagues developed a new technique termed Retrotransposon Capture-sequencing (RC-seq) [232]. This method coupled an array targeting the 5′- and 3′- ends of LINE-1, Alu, and SVA elements with high-throughput sequencing to enrich for potentially rare retrotransposition events. RC-seq revealed that LINE-1, Alu, and SVA retrotransposition had occurred somatically in the human hippocampus and caudate nucleus [232].

More recent work involving whole-genome amplification with RC-seq of single hippocampal neurons revealed almost 14 somatic insertions per cell [233]. Another study from the Walsh group on single cells outside the hippocampus has found a much lower incidence of somatic LINE-1 retrotransposition [234]. We in the field are convinced that LINE-1 retrotransposition is occurring in the brain; however its rate is presently a matter of some controversy. With sensitive methods in place, research over the next 5 years will begin to determine some of the questions research on LINE-1 activity in the brain has generated: [180, 232, 234–238] 1) Are there functional implications for retrotransposition in the brain? 2) Does retrotransposition in the brain contribute to neurological diseases? 3) What is the true rate of retrotransposition in the brain and other somatic tissues?

## Conclusions

LINE-1 and other retrotransposons have moved from mysterious, repetitive sequences in our genome to making appearances in diverse research fields from cancer biology to neuroscience. Future research may reveal that TEs such as LINE-1 are the giant shoulders on which our genome and the cell stands.

## Abbreviations

ADAR: adenosine deaminase acting on RNA
APC: adenomatous polyposis coli
APOBEC3A: apolipoprotein B mRNA editing enzyme catalytic subunit 3A
ASP: antisense promoter
ATM: ataxia telangiectasia mutated
BCC: basal cell carcinoma
C: cysteine-rich
CASP8: caspase 8
CC: coiled coiled
CENPW: centromere protein W
cGAS: cyclic GMP-AMP synthase
CHM: choroideremia
CHO: Chinese Hamster Ovary
CTD: C-terminal domain
CYBB: cytochrome b-245, beta polypeptide
EN: endonuclease
EN_i_: Endonuclease-independent
ERV: endogenous retroviruses
EVE: endogenous viral elements
FKTN: fukutin
F8: factor VIII
HAL1: half-LINE-1
HP1α: heterochromatin protein-1α
KAP1: KRAB-associated protein-1
LINE-1: L1: Long INterspersed Element-1
LTR: Long-Terminal Repeats
MAST2: microtubule associated serine/threonine kinase 2
NF1: neurofibromin 1
NAHR: non-allelic homologous recombination
PRR: pattern-recognition receptor
PGBD5: PiggyBac transposable element-derived protein 5
Poc1a: Poc1 centriolar protein A
PP: processed pseudogenes
PTEN: phosphatase and tensin homolog
RAG1: recombination activating gene 1
RB1: retinoblastoma 1
RC: retrotranspositionally-competent
RC-seq: retrotransposon capture-sequencing
RNaseH: ribonuclease H
RNP: ribonucleoprotein
RPE: retinal pigmented epithelium
RRM: RNA recognition motif
RT: reverse transcriptase
RUNX3: runt related transcription factor 3
SAMHD1: SAM domain and HD domain 1
SINE: Short INterspersed Element
SVA: SINE-VNTR-Alu
SIRT6: Sirtuin-6
SD: splice donor sites
SLCO1B1: solute carrier organic anion transporter family member 1B1
SLCO1B3: solute carrier organic anion transporter family member 1B3
TCF/LEF: T-cell factor/lymphoid enhancer factor
TE: transposable element
THAP9: THAP domain containing 9
TPRT: target-primed reverse-transcription
TREX1: three prime repair exonuclease 1
TSD: target-site duplication
VNTR: variable number tandem repeats
YY1: ying yang 1
